# The Coronaid Program: An App-Based Telemedicine Tool for Triaging, Testing, Treating and Monitoring COVID-19 Patients in Mexico

**DOI:** 10.7759/cureus.19920

**Published:** 2021-11-26

**Authors:** Andrea Rocha-Haro, Dan Morgenstern-Kaplan, Sofía J Canales-Albarrán, Edgar Nuñez-García, Yasshid León-Mayorga

**Affiliations:** 1 Medical Department, Sofía Salud S.A., Mexico City, MEX

**Keywords:** mexico, covid-19, remote care, video-consultation, e-health, telehealth, telemedicine

## Abstract

Objective: To describe the results of a Mexican telemedicine program for patients with coronavirus disease 2019 (COVID-19).

Methods: An observational retrospective study was conducted to analyze and describe the baseline demographic and clinical characteristics of patients who received medical video consultations for respiratory symptoms.

Results: A total of 1,148 video consultations were given from March to September 2020 via Sofía's mobile app. A total of 580 patients sought medical consultation regarding respiratory symptoms. Of the patients, 51% were male and the mean age was 36 years (SD = 13). Of the patients, 35% had comorbidities such as diabetes, hypertension, and obesity, and 1.2% were sent to the ED. Fifty-seven polymerase chain reaction (PCR) tests for COVID-19 were requested and we detected a 53% positivity rate with a mean follow-up of 4.6 consultations.

Conclusion: Telemedicine has proven to be a safe and effective tool for triaging, testing, treating, and remote monitoring of patients with mild COVID-19. Patients triaged by Sofía had good overall outcomes and reduced the risks of in-person consultation in the pandemic.

## Introduction

Telemedicine is defined as the provision of remote clinical services through real-time two-way communication between patients and their healthcare providers, using electronic, audio, and visual means [1]. During the coronavirus disease 2019 (COVID-19) pandemic, telemedicine services have been exponentially increasing due to the inherent risks of in-person consultations and by virtue of telemedicine, all medical services can be provided remotely using technology via video consultations, telehealth platforms, and remote monitoring without risking the healthcare providers [2]. Furthermore, the use of telemedicine for screening patients before in-person visits has proven to be a useful, safe, and cost-effective tool for clinicians in many different medical specialties [3].

Sofía is a Mexican healthcare and technology startup founded in 2018, focused on changing the way patients approach their overall health, by giving them tools such as primary-care video consultations. The initial results of the telemedicine services have been described previously [4]. Since December 2020, Sofía offers a digital health insurance plan that includes a mobile-based telemedicine service that also allows patients to have medical follow-up care with their primary care physicians and referrals with other specialty and subspecialty clinicians, preventive and screening tests, amongst other services.

Mexico is one of the world’s most affected countries by the pandemic. In mid-November 2020, it ranked fourth in recording more than 100,000 COVID-19-related deaths. As of November 2021, more than 4 million Mexicans were affected by this infectious disease and more than 300,000 deaths have been recorded [5]. The pandemic only evidenced an underfunded healthcare system that was already fragile and overstretched, and with the arrival of this challenging coronavirus, the system, as well as all the healthcare workers, collapsed. Based on this context, Sofía decided to launch a program open to the public called “Coronaid” where patients could solicit video consultations with internal medicine specialists for orientation, diagnosis, treatment, and follow-up consultations until discharge.

In this article, we present the outcomes of the general telemedicine approach that was used in Mexican patients with a diagnosis of severe acute respiratory syndrome coronavirus 2 (SARS-CoV-2) infection during the first months of the pandemic.

## Materials and methods

On March 23rd, 2020, in response to the declaration of the beginning of the national emergency period of social distancing by the Mexican Ministry of Health, Sofía implemented its video-consultation service, offering free-of-charge app-based consultations by physicians specialized in internal medicine with expertise in providing remote medical care. The service was initially launched in Mexico City and the metropolitan area, and on April 30, 2020, it was extended to the entire country.

Program development and process

The Coronaid program was developed by Sofía’s medical department, including a guideline for providing remote assessment and treatment in suspected and confirmed COVID-19 cases, based on recommendations from the World Health Organization (WHO), the Centers for Disease Control and Prevention (CDC), and the General Directorate of Epidemiology. The guideline was reviewed by an infectious disease specialist physician.

Through the Coronaid, three different definitions of a suspected case of SARS-CoV-2 infection were used, matching those of the Mexican General Directorate of Epidemiology. The first definition included the criteria of recent traveling to an area with sustained, ongoing community transmission of SARS-CoV-2 [6]. For the second definition, when local transmission of SARS-CoV-2 was reported in Mexico, this criterion was eliminated [7]. The third definition was published on August 25, 2020, changing the days prior to symptoms onset from seven to 10 and the combination of clinical criteria [8].

Once patients were considered as suspect cases of SARS-CoV-2 infection, they were classified in severe and non-severe disease, based on the presence of signs of respiratory distress or oxygen saturation < 90% on room air (measured at home with pulse oximeters). After patients met the criteria defined by the General Directorate of Epidemiology for a suspect case, polymerase chain reaction (PCR) home testing for non-severe cases who lived in Mexico City and the metropolitan area was conducted by Sofía’s medical department and provided by a private laboratory certified by the National Institute of Epidemiological Diagnosis and Reference. PCR results were received via e-mail between 24 and 72 hours after testing. Suspected and confirmed COVID-19 cases with severe symptoms were referred immediately to an emergency department.

**Figure 1:**
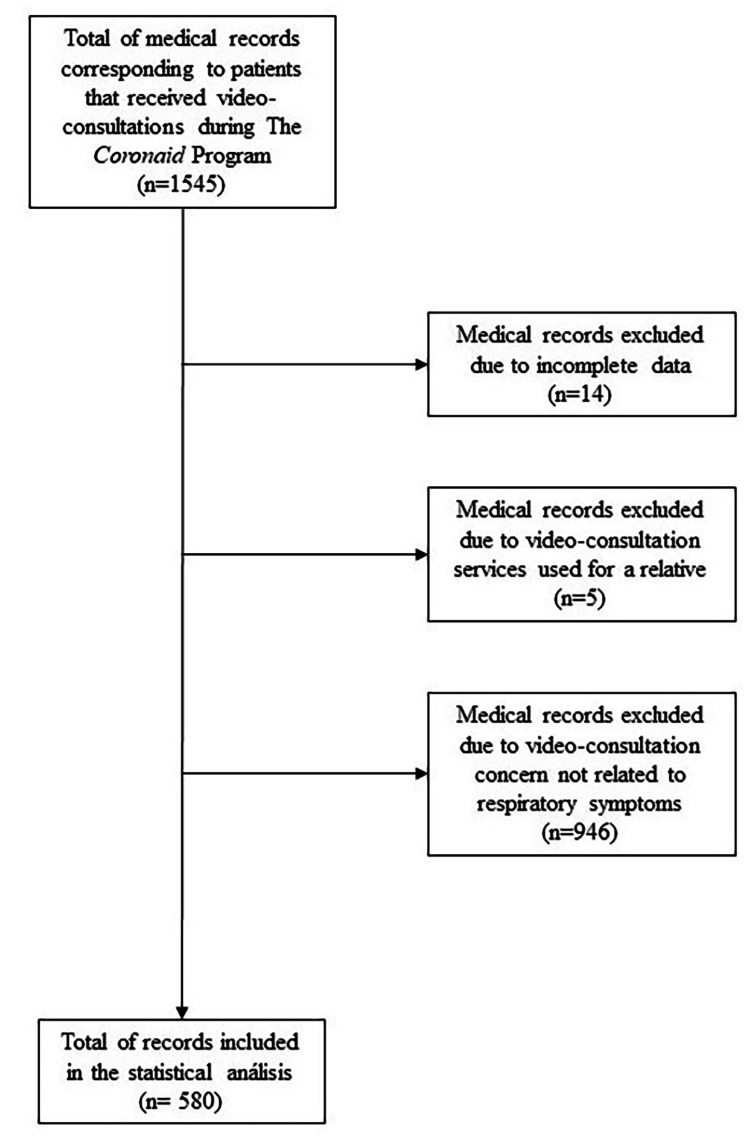
Flow diagram for study participants: selection of medical records at the Coronaid virtual program.

Video consultations were also carried out for patients with an external test, either positive or negative, who were not initially triaged by Sofía’s medical team and classified as suspected cases. Patients received a prescription (which included prescribed medications, alarm signs, and other at-home isolation measures) and indications for a follow-up consultation via the mobile App. Medical data collected in the consultations were registered in Sofía’s electronic medical record. Participants' flow of the telemedicine program is shown in Figure 1.

A website was built for posting relevant information for patients and doctors, including updates of the definition of a suspect case of SARS-CoV-2 infection, hygiene and physical distancing recommendations for the general population, indications for home isolation for suspected and confirmed COVID-19 cases, description of the correct quarantine process for asymptomatic close contacts of confirmed COVID-19 cases, and adequate use of facemasks.

Participants

All patients who were assessed in video consultations via Sofía’s mobile app and who tested positive for SARS-CoV-2 infection (either initially triaged and tested by Sofía’s medical team or requesting medical attention with an external positive test) were included in this study. None of the patients of this study had symptoms of severe disease, they all remained home in self-isolation and received follow-up via video consultation from March 23rd to September 4th, 2020. There were no restrictions regarding age, gender, or comorbidities. Informed consent was obtained for all participants through the mobile application and an Institutional Review Board (IRB) review was waived.

Statistical analysis

All the patients that used Sofía’s video-consultation health services for any respiratory concerns were included in the analysis. Data were collected retrospectively from Sofía’s electronic medical record. We performed a descriptive statistical analysis of the baseline demographic and clinical characteristics of all patients and reported them in frequencies and percentages for nominal and ordinal variables. Means and standard deviations were calculated for numerical variables. Data collection and analysis were performed using the SPSS v. 25 (IBM SPSS Statistics, Armonk, NY) statistical package. Figures were created using R, version 4.0.2 (package “ggplot2”) (R Core Team, Vienna, Austria).

## Results

The Coronaid telemedicine program included on-demand video consultations in the period from March to September 2020 via a mobile app. The global analysis of the 2,585 video consultations that were provided to a total of 1,545 patients has been previously published [4], and here we present the analysis of only the patients with respiratory symptoms during the Coronaid program. A total of 1,148 video consultations were provided through Sofía’s app to 580 patients in relation to any respiratory symptom. Of these patients, 51% were males and 49% were females, and the mean age was 36 years (SD = 13). The mean body mass index was 27 (SD = 5), and 35% of the patients had comorbidities such as diabetes, hypertension, and obesity. Of the patients, 1.2% were referred to an emergency department and only 0.1% had an outcome of death. All baseline demographic characteristics and outcomes are described in Table 1.

**Table 1:** Demographic and clinical characteristics of all patients with respiratory symptoms. SD: standard deviation; BMI: body mass index; PCR: polymerase chain reaction.

Variable	N (%)
Total patients with respiratory symptoms, n (%)	580 (100)
Total video consultations, n	1,148
Male sex, n (%)	292 (51)
Age (years), mean ± SD	36 ± 13
BMI, mean ± SD	27 ± 5
Comorbidities, n (%)	205 (35)
Diabetes, n (%)	33 (5)
Hypertension, n (%)	60 (10)
Obesity, n (%)	138 (23)
PCR test, n (%)	146 (25)
Sofia’s PCR test, n (%)	57 (10)
External PCR test, n (%)	89 (15)
Smoking, n (%)	100 (17)
Emergency room indication, n (%)	7 (1.2)
Deaths, n (%)	1 (0.1)

During the video-consultation assessment, the criteria for a suspected case of COVID-19 established by the national epidemiology department were applied (defined and referenced in the methods section). If the patient met these criteria, the request for a PCR test was submitted. A total of 57 tests were solicited through Sofía's app, while 89 patients arrived with an external test to receive guidance for treatment and medical follow-up. Clinical characteristics of tested patients can be found in Table 2.

**Table 2:** Baseline and clinical characteristics of patients tested for SARS-CoV-2 infection. SARS-CoV-2: severe acute respiratory syndrome coronavirus 2; PCR: polymerase chain reaction; BMI: body mass index.

Variable	Sofia’s tested patients	External tested patients
Total of PCR tests, n (%)	57 (100)	89 (100)
Positive PCR test, n (%)	30 (53)	81 (91)
Median follow-up (consultations)	4.67	2.45
Male sex, n (%)	14 (47)	43 (48)
Age (years)	37 ± 13	37 ± 13
BMI, mean ± SD	27 ± 3	27 ± 4
Comorbidities, n (%)	12 (40)	38 (43)
Diabetes, n (%)	2 (7)	4 (5)
Hypertension, n (%)	5 (17)	12 (14)
Obesity, n (%)	8 (27)	30 (34)
Overweight, n (%)	10 (33)	30 (34)
Smoking, n (%)	10 (33)	10 (11)
Emergency room indication, n (%)	0	7 (7.8)

**Figure 2:**
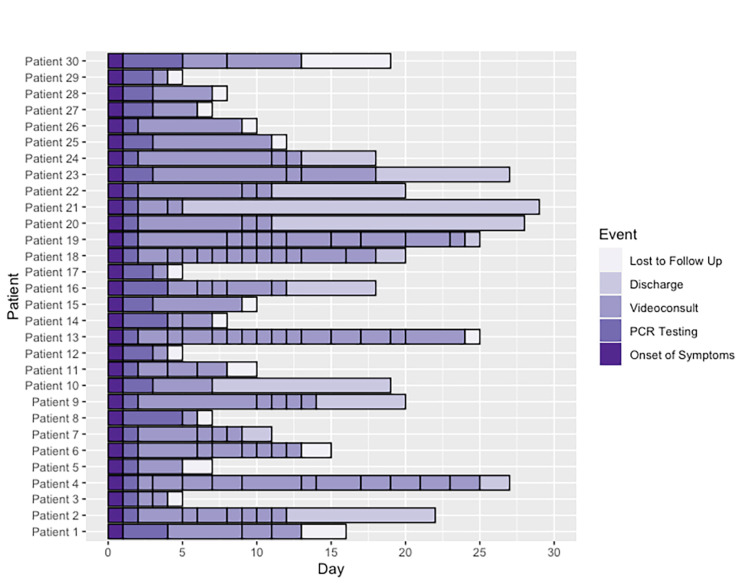
Timeline of the 30 patients with SARS-CoV-2 infection triaged by video consultation. This timeline graph represents the clinical progression of the 30 patients who were triaged and diagnosed with COVID-19 through Sofía's PCR testing. Each black line represents a contact through video consultations and the time from onset of symptoms to discharge of loss of follow-up. COVID-19: coronavirus disease 2019; SARS-CoV-2: severe acute respiratory syndrome coronavirus 2; PCR: polymerase chain reaction.

**Figure 3:**
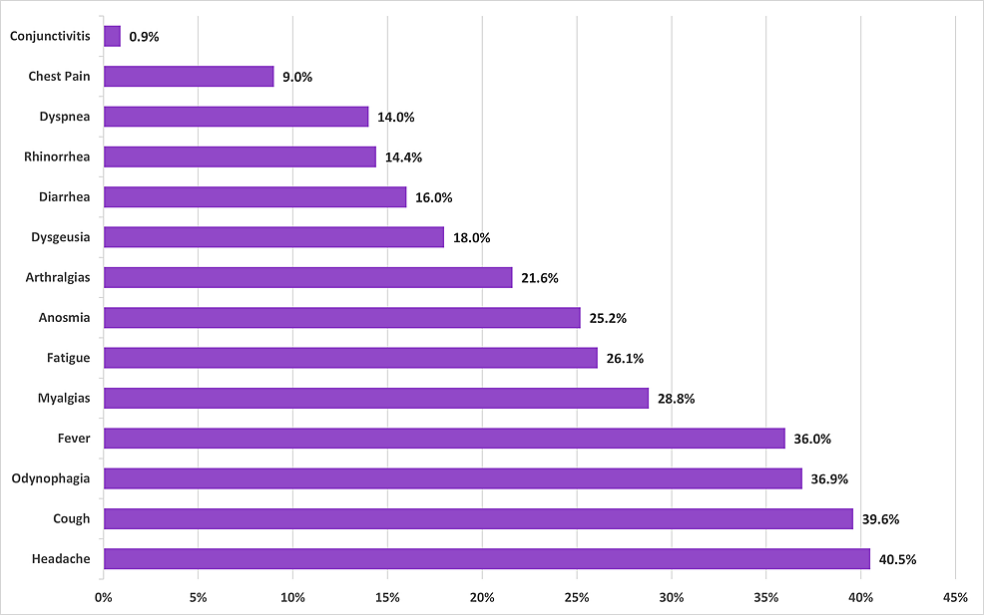
Distribution of symptoms (%) among all patients with a positive PCR test for SARS-CoV-2. PCR: polymerase chain reaction; SARS-CoV-2: severe acute respiratory syndrome coronavirus 2.

Of the 57 PCR tests solicited by Sofía's physicians, 53% were positive. The mean follow-up per patient was 4.6 video consultations vs. 2.45 in the group of external PCR tests who did not receive an initial assessment by Sofía's physicians. In this last group of patients, seven people were required to go to the emergency room, representing 7.8% of this population with an external test in contrast to no patients in the internal testing group. The clinical progression and video-consultation frequency of the 30 patients with COVID-19 diagnosed through Sofía's testing is described in Figure 2. The most frequently reported symptoms among patients with a positive PCR test for COVID-19 are displayed in Figure 3.

## Discussion

The COVID-19 pandemic led to the implementation of virtual medical care programs all around the world that provided less person-to-person contact, enhancing patients’ safety by not having to leave their homes to seek medical attention. Some programs included patient monitoring either through video consultation or phone calls and some even designed platforms for patient education with specific COVID-19 content that also included forms for inquiries about current symptoms [9].

A program developed in Minnesota used a previously adopted platform that was designed for postoperative patient follow-up. Virtual care by medical providers was available 24 hours a day, seven days a week, and symptoms were color-flagged based on the severity. They triaged patients and determined the next level of care, which included either an emergency department visit, urgent care, video-clinic visit, or phone encounter. The number of patients attended (n = 1,496) as well as the median age (38 years old) were remarkably similar to that of our program, except for gender distribution with a female predominance (62%) and an average of three interactions with the platform per patient. Only 8% of their patients had a COVID-19 test, of which only 1% were positive and 13 patients admitted to the hospital were reported [10].

Another virtual outpatient program was developed at the Sunnybrook Health Sciences Centre in Toronto, Ontario, to provide medical care for recently COVID-19 diagnosed patients. A total of 50 patients were assessed and they observed that most people presented with a mild severity illness. The median days from symptom onset to healthcare assessment were three days. Only four patients were sent and admitted to the hospital, and the median age of these patients was 61 years old. They had a coordinated process in case of clinical deterioration requiring hospital transfer. Patients' demographic characteristics were not similar to those observed in our program but treating them remotely also helped diminish the healthcare system burden and was associated with good outcomes [11].

Regarding our program, the positivity rate was 53% for patients who were triaged by Sofía’s medical team and tested at home (n = 57) through epidemiological weeks 17 to 27, similar to the mean national rate of 48.54% and the 47.73% reported in Mexico City in the same period [8]. All 57 patients were considered suspect cases using the same definition [6].

The clinical presentation of our patients with COVID-19 was similar to other case series reported in most symptoms (cough, odynophagia, fatigue, myalgia, and arthralgia); however, our patients reported headache as the most prevalent symptom (40.5%), while other studies report its prevalence as lower than the present study [12]. The frequency of fever in our patients was inferior to that reported in other studies [13]. The low frequency of severe symptoms in this cohort could be associated with the fact that most patients were outpatients and received telemedical triage to assess the severity of symptoms and possible referral for hospital admission, which further adds to the fact that telemedicine is a useful and effective tool for clinicians to determine if patients require hospitalization or ambulatory care.

## Conclusions

Telemedicine initiatives may have many disadvantages such as increased costs for purchasing equipment and technical training for physicians and caregivers to ensure an effective telemedicine program, and in cases where patients use an on-demand consultation service, it may be with a different healthcare provider, therefore, reducing care continuity. Fortunately, telehealth services are associated with major advantages such as lowering healthcare costs derived from transportation expenses and unnecessary non-urgent emergency department visits, increasing medical care efficiency, and better access to healthcare services for patients who are homebound or who live in remote locations. Finally, during the ongoing COVID-19 pandemic, telemedicine has served as an invaluable tool to extend care, closely monitor disease progression, and timely referrals to emergency departments for millions of patients worldwide. Although the Coronaid was a time-limited program, telemedicine services continue to grow throughout the world, including in Mexico.

Telemedicine has led to the insight of a new approach to medical consultation and represents a challenge for medical providers. Medical assessment, diagnosis, and treatment can be effectively practiced through the adoption of virtual software platforms, well-based remote care programs, and appropriately trained physicians and patients. Virtual medical care should be practiced not just as a temporary modality during the pandemic but continuously to disrupt the inaccessibility of health care present among some populations, especially vulnerable groups and rural communities.
